# Hidden barriers to leadership: a cross-sectional survey of prevalence and predictors of Imposter Phenomenon in Trauma and Orthopaedic surgery in the UK

**DOI:** 10.1136/bmjopen-2025-100557

**Published:** 2025-09-11

**Authors:** Zaha Kamran Siddiqui, James Tomlinson, Arabella Scantlebury, Raveen Jayasuriya, Helen Church, Amy Grove

**Affiliations:** 1 Manchester University NHS Foundation Trust, Manchester, UK; 2 Sheffield Teaching Hospitals NHS Foundation Trust, Sheffield, UK; 3 The University of Sheffield Faculty of Medicine Dentistry and Health, Sheffield, UK; 4 Centre for Evidence and Implementation Science, University of Birmingham, Birmingham, UK; 5 University of Nottingham, Nottingham, UK

**Keywords:** ORTHOPAEDIC & TRAUMA SURGERY, Social Interaction, MEDICAL EDUCATION & TRAINING

## Abstract

**Abstract:**

**Objectives:**

Imposter Phenomenon is characterised by persistent self-doubt despite objective success. It has been associated with anxiety, burnout and reduced job satisfaction. Little is known about imposter phenomenon’s presence and impact in Trauma and Orthopaedic surgery. This study aims to determine the prevalence and predictors of Imposter Phenomenon among UK orthopaedic surgeons, further mapping domains that affect leadership and professional development.

**Design:**

Cross-sectional survey using the validated Clance Imposter Phenomenon Scale (CIPS).

**Setting:**

The survey was distributed to UK orthopaedic surgeons between 20 October 2023 and 28 February 2024 via Training Programme Directors and the British Orthopaedic Association.

**Participants:**

Orthopaedic trainees and consultant surgeons (n=441)

**Primary and secondary outcome measures:**

Imposter Phenomenon severity measured using CIPS (mild: 41–60, moderate: 61–80 and severe: 81–100). Univariate and multivariate analyses identified predictors of this severity. Self-reported impact of Imposter Phenomenon assessed across personal and leadership domains.

**Results:**

92% of respondents reported moderate to intense Imposter Phenomenon symptoms (mean CIPS=65.17). Trainees had significantly higher mean scores (70.64±13.85) compared with consultants (59.82±15.71). Female surgeons reported significantly higher mean scores (72.57±13.35) than male surgeons (61.19±15.74). Female gender, non-consultant training grade and time out of training were predictors of severity (p<0.01). 90% reported negative impacts, with 49% discouraged from applying for leadership roles and 45% experiencing hindered career progression.

**Conclusion:**

The Imposter Phenomenon is highly prevalent among UK orthopaedic surgeons; disproportionately affecting women, trainees and those taking career breaks. Imposter Phenomenon significantly impacts leadership aspirations and career development, potentially contributing to reduced diversity in surgical leadership. Targeted interventions addressing Imposter Phenomenon are needed to support equitable leadership development in Trauma and Orthopaedic surgery.

**STRENGTHS AND LIMITATIONS OF THIS STUDY**

- The validated Clance Imposter Phenomenon Scale, with high internal consistency (Cronbach’s alpha 0.85–0.96), was used to ensure standardised measurement of Imposter Phenomenon severity.
- Survey distribution through Training Programme Directors, LinkedIn and the British Orthopaedic Association facilitated broad recruitment and reduced selection bias.
- Self-reported measures of Imposter Phenomenon and its impact may be influenced by social desirability bias.
- The cross-sectional design limits the ability to establish causality between predictors and Imposter Phenomenon severity.
- Higher-order demographic groupings were required due to small subgroup sizes, potentially masking more granular variations.

## Introduction

 Imposter Phenomenon (IP) is a pattern of thinking affecting high-achieving individuals across various professions, manifesting as pervasive self-doubt and fear of being exposed as a fraud despite clear evidence of success and competence.^1^ Initially identified by Clance and Imes in high-achieving women, IP has since been recognised as a phenomenon that impacts all genders.^2^ A growing body of research highlights significant gender disparities in IP experiences within surgical specialties. However, the association between gender and IP findings remains inconclusive.^3^,^5^ Male participation in IP interventions is limited,^6^ perpetuating the gendered perception of IP. This reluctance to participate limits awareness of IP and reduces acknowledgement of its effects on peers, thereby perpetuating its consequences in the wider workplace.

Surgical training, particularly in the UK, demands early and sustained commitment to exams and research activities, fostering a culture of constant comparison, high performance and perfectionism.^7^ Perfectionism is also associated with IP.^2 8^ Perfectionist tendencies may lead surgeons to focus on mistakes over achievements, fuelling self-doubt. These pressures, combined with hierarchical feedback and limited psychological support, can exacerbate IP across all training stages.

The field of Trauma and Orthopaedics (T&O) presents unique cultural dynamics that amplify existing pressures. Historically, T&O has been a male-dominated field, with only 11% of the British Orthopaedic Association (BOA) membership being female.^9^ This culture has been shaped by hierarchical training environments and expectations of physical stamina and technical expertise, reinforcing traditional ideals of surgical toughness.^10^ These cultural norms may also contribute to under-representation of women and minorities, creating additional pressures to conform and excel for those outside the traditional archetype. The limited representation of women and ethnic minorities within T&O further exacerbates IP, as these groups may feel the need to demonstrate exceptional performance to overcome stereotypes and prove their worth in a historically male-dominated and predominantly white specialty.^11^

IP has been extensively studied among medical students and speciality trainees; however, much of this research has originated in the USA.^2^ Characterisation of IP in surgical specialities remains limited and is similarly concentrated in the USA, focusing on fields such as Plastics,^12^ General surgery,^3^ Obstetrics and Gynaecology,^13^ Maxillofacial Oral,^14^ Paediatrics^15^ and Orthopaedics.^16^ Notably, only one study to date has examined IP in Neurosurgery, conducted in Italy.^17^ This highlights a significant gap in the exploration of IP in surgical trainees and consultants within surgical specialties in the UK.

IP poses risks to individual mental health leading to depression, anxiety, burnout and suicidal ideation.^2 18^ IP negatively affects career progression by fostering self-doubt,^19^ which discourages individuals from pursuing opportunities like promotions or leadership roles, threatening institutional diversity.^20^,^22^ This may be particularly problematic for women and ethnic minorities in male-dominated fields like T&O, where systemic barriers already limit representation. Inherently, IP may affect diversity and leadership, which are critical for innovation and driving better patient outcomes.^9 23^ Furthermore, IP can impact communication, decision making and problem solving in surgeons.^24^ These are key non-technical factors required by all surgeons to lead their day-to-day clinical practice. Although studies have examined anxiety^13^ and burnout^4^ in surgeons experiencing IP, the wider impact of IP on surgeons’ personal and professional lives and its influence on surgical leadership remains unexplored. Therefore, as a secondary goal, the study aims to map the impact of IP in surgeons and highlight leadership domains that might be affected.

Exploring IP in T&O is essential to understand its prevalence and examine its broader implications for clinical practice and leadership. This study seeks to address three critical gaps in the literature:

1. What is the prevalence of IP among T&O surgeons in the UK?
2. What factors predict increased severity of IP within the field of T&O?
3. How does IP impact T&O surgeons, particularly in the context of leadership and professional development?

## Methods

### Study design and setting

A cross-sectional survey was conducted using Google Forms (Google LLC), with ethical approval granted by the University of Sheffield (ref:056897). The survey was distributed by orthopaedic training programme directors across the UK. To address potential sources of bias, survey distribution was broadened through multiple platforms, including LinkedIn and the BOA, ensuring diverse outreach. Data collection took place over a span of 4 months, from 20 October 2023 to 28 February 2024. No formal sample size calculation was performed due to the exploratory nature of the study.

### Participants

The study included T&O surgical trainees, Speciality and Associate Specialist (SAS), post Certificate of Completion of Training (CCT) fellows and consultants actively practising in the UK. Medical students, foundation trainees and those practising abroad were excluded.

### Patient and public involvement

Patients and the public were not involved in the design, conduct, reporting or dissemination of this study.

### Variables

The survey had three sections: (1) participant demographics; (2) Clance IP Scale (CIPS) questionnaire^25^ and (3) a self-reported assessment of IP’s impact on personal and professional domains. The primary outcome was the severity of IP, measured using the CIPS questionnaire.^25^ The CIPS assesses feelings of self-doubt, fear of being exposed as a fraud and internalisation of success as unearned, with scores categorised into mild (41–60), moderate (61–80), and severe (81–100) IP levels. Predictors included demographic variables: gender, ethnicity, training grade and time out of training.

### Data sources/measurement

CIPS is a 20-item questionnaire rated on a 1–5 scale, with Cronbach’s alpha values ranging from 0.85 to 0.96, indicating high internal consistency.^25^ To reduce missing data, all CIPS survey questions required completion. Each item appeared individually on-screen to minimise response anchoring, encouraging more honest and independent reflections.^26^ The impact assessment was designed based on a review of the existing literature to identify leadership and development domains affected by IP in healthcare. Participants were asked to select all applicable impact domains from a predefined list, which included self-esteem, career progression, decision-making, work-life balance and others (table 4).

### Analysis

Continuous variables are reported as mean values with SD and ranges, while categorical variables are presented as frequencies and percentages.

To account for small sample sizes and to ensure a statistically meaningful analysis, higher-order groupings were applied to both training phase and ethnicity. UK training grades were initially organised into training phases as per the Intercollegiate Surgical Curriculum Programme: phase 1 (core training (CT)/speciality training (ST) 1–2), phase 2 (ST3–6), phase 3 (ST7–8), post-CCTfellows, consultants and SAS surgeons. However, due to the small number of respondents in phases 1 (n=11), phase 3 (n=39) and post-CCT fellows (n=29), training grades were grouped into two main categories prior to statistical analysis: consultants and non-consultants (ST1-8 and post-CCT fellows). Since SAS surgeons typically hold unique roles and responsibilities that overlap with those of both training-grade and consultant surgeons, they were considered a separate category and excluded from the overall statistical analysis due to low responses (n=6).

For ethnicity, participants were grouped into the following categories: people of colour (POC) (Arab, Pakistani, Chinese, Indian, Vietnamese, Malaysian, Bangladeshi, Caribbean, Sri Lankan, Asian other, African); white (English, Scottish, Irish, Welsh, White African, European, White mixed, White Northern Irish, White other) and other (Coptic, South African, Australian, New Zealand). These groupings were self-imposed based on the need for broader categories to facilitate analysis, as the majority of the categories had numbers of three or fewer.

Statistical analyses were conducted using IBM SPSS Software (V.29.0.2.0). Univariate analysis was performed using the Mann-Whitney U test and Kruskal-Wallis test to evaluate differences in CIPS scores by gender, ethnicity, training grade and time out of training, with a p value threshold of <0.05 for statistical significance. The Mann-Whitney U test was used for two-group comparisons (gender: male/female and time out of training: yes/no), while the Kruskal-Wallis test was used for multigroup comparisons, with ethnicity as the grouping variable with three categories (POC, white, other) and training grade as the grouping variable with 2 categories (consultants, non-consultants). Additionally, a multiple linear regression analysis was conducted to assess potential predictors of IP severity. The predictors included demographic variables: consultant status, gender, ethnicity and time out of training. Multicollinearity diagnostics were performed to ensure independence of predictor variables.

## Results

In comparison to the most recent BOA membership data which includes 5195 members, our survey received 447 responses (response rate: 8.5%).^9^ Of these, 441 responses were considered for analysis, as six surgeons were not UK-based. Participant characteristics are summarised in table 1. The majority of the respondents were white (69%), male (64%) and consultant T&O surgeons (51%) based at tertiary/university teaching hospitals or major trauma centres (59%). The distribution of participants across regions of training shows a wide geographical spread, with the largest group trained in the Yorkshire & Humber (20%), followed by Northwest of England (13%) and West Midlands (12%). Among the white participants (n=305), 71% identified as white English, while the most prevalent ethnicity among POC participants was Asian Indian (46%).

**Table 1:** Demographic data of surveyed respondents

Characteristic	Mean (SD)	Range
Birth year	1982 (9)	1954–1998
Year of graduation	2006 (11)	1900–2021
Overall CIPS score	65.17 (15.76)	21–97

	Total number of respondents (n=441)	Percentage of respondents (%)
Training grade		
Phase 1: CT/ST1–2	11	2
Phase 2: ST3–6	133	30
Phase 3: ST7–8	39	9
Post CCT fellow	29	7
SAS surgeons	6	1
Consultants	223	51
Region of training		
East of England	19	4
KSS	34	8
London	27	6
Midlands (east)	23	5
Midlands (west)	53	12
Northeast England	29	7
Northwest England	58	13
Northern Ireland	9	2
Scotland	43	10
Southwest England	17	3.8
Thames Valley	12	3
Wales	17	3.8
Wessex	8	2
Yorkshire & Humber	90	20
Prefer not to say	2	0.4
Type of Hospital		
Tertiary/university teaching hospital/major trauma centre	259	59
District general	174	39.6
Private	2	0.4
On leave	6	1
Gender		
Male	284	64
Female	154	35
Prefer not to say	3	0.7
Ethnicity		
White	305	69
POC	108	25
Other	23	5
Prefer not to say	5	1
Time out of training		
Yes	203	46
No	238	54
IP category (score)		
Mild (≤40)	37	8
Moderate (41–60)	126	29
Frequent (61–80)	199	45
Intense (>80)	79	18

CCT, Certificate of Completion of Training; CT, core training; IP, imposter phenomenon; KSS, Kent, Surrey and Sussex; POC, people of colour; SAS, Speciality and Associate Specialist; ST, speciality training.

### Prevalence and predictors of IP among T&O surgeons

Most T&O surgeons (91.6%) reported moderate-intense levels of IP. Notably, 45% of respondents reported *frequent* imposter symptoms and nearly one in five (18%) fell into the *intense* category, highlighting the high prevalence of severe IP in T&O.

Our study identified significant differences in the prevalence of IP based on gender (p<0.001), training grade (p<0.001) and time out of training (p<0.001). No significant difference was found across ethnicity and IP scores (p=0.34) (table 2).

**Table 2:** CIPS categorisation by demographic and professional characteristics

	IP category score (n)				Overall n (%)	Mean CIPS Score (SD)	P value
	Mild (≤ 40)	Moderate (41–60)	Frequent (61–80)	Intense (>80)			
Overall	37	126	199	79	441 (100)	65.17 (15.76)	
Gender							<0.001
Female	3	24	77	50	154 (35)	72.57 (13.35)	
Male	34	100	121	29	284 (65)	61.19 (15.74)	
Ethnicity							
POC	4	40	52	12	108 (26)	64.07 (13.85)	0.34
Non-POC	32	73	138	62	305 (69)	65.68 (16.42)	
Other	0	11	7	5	23 (5)	65.65 (15.17)	
Training grade							
Consultants	30	84	91	18	223 (51)	59.82 (15.71)	<0.001
Non-consultants	7	42	108	61	218 (49)	70.64 (13.85)	
CT/ST1-2	0	4	4	3	11 (2)	71.18 (12.97)	
ST3-6	6	24	65	38	133 (30)	70.42 (14.49)	
ST7-8	1	7	21	10	39 (9)	70.15 (12.28)	
Post CCT-fellow	0	4	15	10	29 (7)	74.45 (13.19)	
SAS	0	3	3	0	6 (1)	59.17 (8.47)	
Time out of training							
Yes	11	44	108	40	203 (46)	68.24 (14.52)	<0.001
No	26	82	91	39	238 (54)	62.55 (16.33)	

CIPS, Clance Imposter Phenomenon Scale; CT, core training; IP, imposter phenomenon; POC, people of colour; SAS, speciality and associate specialist; ST, speciality training.

Surgeons in training reported significantly higher mean CIPS scores (70.64) compared with consultants (59.82), p<0.001. From CST1 to post-CCT fellow, imposter scores remained consistently elevated. Consultants reported a lower mean CIPS score; however, the majority of consultants (n=91) fell into the *frequent* category.

Female surgeon respondents demonstrated significantly higher mean CIPSs (72.57) compared with their male counterparts (61.19), p value <0.001. *Frequent* levels of IP were common in both genders (table 2); however, a larger number of female surgeons reported intense IP (n=50) compared with male surgeons (n=29). Additionally, more male surgeons experienced IP at mild levels (n=34) compared with female surgeons (n=3), highlighting a gendered pattern in the experience of IP. In total, 45% (n=197) of respondents reported having taken time out of training, primarily for maternity/paternity leave (n=67) and 9% took time out of training more than once (table 3). Although most surgeons in each group demonstrated *frequent* IP (table 2), those who had taken time out of training had significantly higher mean CIPS scores than those who had not (68.24 vs 62.55, p<0.001).

**Table 3:** Overview of respondents (n=441) who reported taking time out of training, categorised by the purpose of their time out

	No. of total respondents (%)
Time out of training	
No	244 (55)
Yes	197 (45)
Breakdown of time out of training	
Maternity/paternity leave	67 (34)
Extended sick leave	37 (19)
Out of programme research	37 (19)
Locum post	30 (15)
Out of programme training	25 (13)
Out of programme clinical experience	25 (13)
Out of programme career break	13 (7)
Sabbatical	5 (2)
Out of programme, out of programme pause	3 (1)

A multiple linear regression was conducted to assess predictors of higher CIPS scores in this professional group. Consultant status (B=−9.939, p<0.001) and male gender (B=−8.298, p<0.001) were a significant negative predictor of high CIPS score. Further, those who took time out of training had a positive effect on CIP scores (B=3.013, p=0.034). Ethnicity was not a predictor of severity of IP (B=1.277, p=0.338). The analysis revealed no significant multicollinearity issues.

### Impact of IP

Among the 441 respondents, 90% reported experiencing negative impacts from IP. A small proportion (10%) reported no effects of IP, with most of these individuals having *mild* IP scores (n=27, 6%). Further, the most affected domain was self-esteem, as reported by 66% of participants who reported being negatively impacted by IP (table 4).

**Table 4:** Impact of IP on T&O surgeons in the UK stratified by severity reveals varied effects across both professional and personal domains

Impact of IP	Total responses (n=441) (%)	CIPS category			
		Mild (n=37) (%)	Moderate (n=126) (%)	Frequent (n=199) (%)	Intense (n=79) (%)
Not impacted by IP	46 (10)	27 (6)	16 (3)	2 (0.4)	1 (0.2)
Impacted by IP	395 (90)	10 (2)	110 (25)	197 (45)	78 (18)

Impact domain	Total impacted (n=395) (%)	Mild (n=10) (%)	Moderate (n=110) (%)	Frequent (n=197) (%)	Intense (n=78) (%)
Self-esteem and self-worth	262 (66)	0 (0)	55 (50)	139 (70)	68 (87)
Applying to leadership roles	193 (49)	1 (10)	51 (46)	99 (90)	42 (54)
Career progression	179 (45)	2 (20)	43 (39)	99 (90)	35 (45)
Decision making	174 (44)	2 (20)	30 (27)	97 (88)	45 (58)
Mental/emotional well-being	167 (42)	0 (0)	27 (24)	87 (79)	53 (68)
Work-life balance	165 (41)	2 (20)	33 (30)	87 (79)	43 (55)
Pursuit of new opportunities	149 (38)	1 (10)	30 (27)	77 (70)	41 (53)
Relationships with colleagues	145 (37)	2 (20)	32 (29)	77 (70)	34 (44)
Skill development and learning	145 (37)	1 (10)	25 (23)	76 (69)	43 (55)
Job satisfaction	140 (35)	2 (20)	15 (14)	74 (67)	49 (63)
Financial aspects (eg, negotiating salary)	106 (27)	0 (0)	28 (25)	58 (53)	20 (26)
Goal setting/achievement	95 (24)	1 (10)	16 (15)	50 (45)	28 (36)
Communication	56 (14)	0 (0)	15 (14)	29 (26)	12 (15)
Physical health	43 (11)	0 (0)	4 (4)	20 (18)	19 (24)

CIPS, Clance Imposter Phenomenon Scale; IP, imposter phenomenon; T&O, trauma and orthopaedics.

The survey revealed that IP significantly impacts T&O surgeons across various domains critical to leadership and professional development. Nearly half of the respondents (49%) impacted by IP indicated that it discouraged them from applying to leadership roles, with this effect being particularly pronounced among female surgeons (49%) and those with frequent (90%) and intense (54%) IP. Similarly, 45% reported that IP hindered their career progression, while 38% indicated reluctance to pursue new opportunities due to self-doubt. Decision-making, a key leadership competency, was affected in 44% of respondents, particularly those with frequent (88%) and intense (58%) IP.

We found that IP also negatively influenced skill development and learning in 37% of respondents. Furthermore, job satisfaction (35%) and work-life balance (41%) were adversely affected, highlighting broader implications for sustained professional growth.

## Discussion

This study aimed to evaluate the prevalence of IP among UK-based T&O surgeons, identify factors contributing to severity of IP and map the impact of IP on domains of leadership and professional development. Our study marks the first comprehensive assessment of IP within T&O surgery in the UK, based on a sample size of 441 respondents. Our findings demonstrate that IP is highly prevalent among UK T&O surgeons, with 91.6% experiencing moderate to intense levels of IP and a mean CIPS score of 65.17.

Recent data from a USA-based study in Orthopaedic residents provides direct comparison opportunities, reporting 73% prevalence of significant or intense IP.^16^ Our frequent and intense categories combined (63%) align closely with these figures, suggesting consistent patterns of IP across healthcare systems. Our reported higher prevalence of 91.6% reflects the inclusion of moderate symptoms, which were not emphasised in the US study’s primary analysis. This comparison highlights how excluding moderate symptoms may lead to underrepresentation of IP’s true scope in the literature.

The predictors of IP severity in our study included female gender, non-consultant training grade and career interruptions. Interestingly, while studies have identified ethnicity as a factor influencing IP,^2^ our analysis found no significant effect of ethnicity on IP. The self-reported impact of IP extended to self-esteem, career progression, decision-making and reluctance to pursue leadership opportunities, aligning with findings in the wider literature on the detrimental effects of IP in healthcare.

### Gender disparity in IP

The findings of this study add to the growing literature highlighting significant gender disparities in IP experiences within surgical specialties. The high-stakes nature of surgery fosters perfectionist tendencies and ongoing performance scrutiny, which may exacerbate self-doubt.^8 27^ Women in surgery often face societal pressures that heighten these feelings, contributing to diminished self-assertiveness and lower self-esteem.^27 28^ Previous research offers mixed evidence on gender differences in IP among surgeons, with some studies showing no significant disparities, while others report higher prevalence among female surgical trainees compared with their male peers.^3^,^5^

Our finding that female T&O surgeons have significantly higher CIPS scores (72.57 vs 61.19, p<0.001) is remarkably consistent with a recent US-based study identifying female orthopaedic residents were 5.64 times more likely to experience significant or intense IP.^16^ This cross-national consistency suggests that gender disparities in IP within orthopaedic surgery transcend healthcare systems and may reflect deeper structural and cultural issues within the speciality globally. Evidence suggests that women may feel compelled to achieve a higher level of competence before expressing confidence in their abilities, while men may project confidence earlier, even before they are fully skilled.^29^ Studies on IP in women outside of medicine often show no significant gender differences,^2^ underscoring the critical role of workplace environments and social support systems in shaping these experiences.^30^ The gendered nature of IP in surgery, particularly in hierarchical male-dominated specialties like T&O, includes ingrained gender biases, limited representation of women in senior leadership roles and the challenge of balancing career demands with societal expectations.^31^ These factors may contribute to the elevated experiences of IP among female orthopaedic surgeons, as they navigate these systemic barriers while striving to meet high professional standards.

IP has been stereotypically viewed as a predominantly female concern—evidenced by the fact that the majority of IP interventions have limited male participation.^6^ Cultural norms surrounding masculinity and confidence often discourage men from openly acknowledging self-doubt,^29^ perpetuating the silence around their experiences with IP. This reluctance can further isolate male surgeons who may be grappling with these feelings, while also limiting their ability to empathise with the unique challenges faced by their female colleagues. Growing evidence, including our findings that 88% of male T&O surgeons reported moderate to intense IP, indicates that male surgeons experience it in significant numbers as well.^5^ By acknowledging their own vulnerabilities, male surgeons can help break down the stigma surrounding IP. Encouraging male surgeons to engage in IP-related discussions and interventions not only challenges the stereotype of IP as a gender issue but also reinforces the importance of peer and institutional strategies in combating its negative impacts.^6^

### IP in surgical training

T&O surgical trainees are particularly susceptible to high IP scores due to the competitive and high-pressure nature of their training.^7^ From the outset, they face intense pressure to excel academically, technically and professionally, which can foster feelings of self-doubt. The hierarchical structure of surgical training, with frequent public feedback and critical evaluations, may exacerbate these feelings, as trainees internalise criticism and question their competencies.^32^ Moreover, trainees often undergo transitional phases, such as moving from junior to senior roles, which can trigger uncertainty and reinforce IP. The lack of psychological support during training further intensifies these challenges, leaving trainees vulnerable to isolation and self-doubt.^28^ The constant comparison with peers also contributes to a sense of inadequacy, particularly when trainees perceive others as more competent. These factors create an environment where IP is prevalent, making it crucial to implement support systems to address both technical and psychological challenges during surgical training.

### Time out of training

Our study is the first to identify time out of training as a significant predictor of IP severity among T&O surgeons. Transitioning into a new stage of practice or returning from a career break heightens self-doubt, as individuals may feel compelled to project confidence even when they do not fully feel it.^19^ Clinical and professional skills can decline over time when not regularly practised,^33^ which presents unique challenges for T&O surgeons returning to practice after a break. The speciality is highly craft-based, and maintaining technical proficiency requires continuous practice of complex procedures. Although programmes like the Supported Return to Training (SuppoRTT) are designed to assist in reintegrating surgeons back into practice, those prone to feelings of IP may experience additional pressure to prove their abilities.^34^ This may further exacerbate feelings of self-doubt.

Transitions, such as returning after a break, can serve as valuable intervention points for targeted strategies to challenge IP. Normalising the temporary dip in confidence that many surgeons experience during transitions is crucial, and openly communicating that this phase is both common and temporary can reduce feelings of self-doubt. By integrating IP-focused workshops into the existing SuppoRTT programmes, institutions can create awareness about expected challenges.

### Impact of IP on T&O surgeons

Existing literature highlights that imposter feelings undermine confidence, decision-making and the pursuit of career advancement opportunities, all of which are critical to developing leaders in surgery.^13^ Self-doubt and self-esteem were hindered in more than half of our respondents. Evidence suggests that self-esteem can in turn affect performance and job satisfaction.^29 35^ Our findings demonstrate the pervasive impact of IP on leadership aspirations, with nearly half of respondents indicating reluctance to pursue leadership roles due to IP. This reluctance was particularly pronounced among female T&O surgeons and those with *frequent/intense* IP, suggesting that IP may contribute to the under-representation of women in senior surgical roles, further threatening institutional diversity.^30^ If surgeons who aspire to be leaders avoid these roles due to IP, healthcare institutions may miss out on diverse perspectives and leadership styles essential for innovation and effective team dynamics.^20 21 30^ Additionally, under half of the respondents reported IP negatively impacted their career progression and decision-making abilities. A recent study reports increased intolerance of uncertainty and lower problem-solving confidence with higher levels of IP in orthopaedic surgeons.^24^ Self-doubt fostered by IP may contribute to hesitation or even reluctance to make independent decisions, further affecting leadership in surgeons.

IP significantly disrupted work-life balance and adversely affected mental health among T&O surgeons. Burnout, characterised by emotional exhaustion and depersonalisation, has been well-documented in surgical specialties, particularly in T&O in the UK.^36^ Many affected surgeons report symptoms of anxiety and depression linked to persistent feelings of inadequacy.^2 18^ The link between IP and burnout is particularly concerning, as it not only compromises the well-being of surgeons but also threatens professional development, team dynamics and patient care.^4^

Interventions could be tailored to help surgeons manage self-doubt and reframe their perceptions of failure, supporting individual careers while also ensuring the sustainability and inclusivity of the surgical workforce.

### Limitations

Our cross-sectional design captured data at a single time point, which limits our ability to establish causality between factors, such as time out of training and elevated IP severity.

The use of higher-order groupings for ethnicity and training phase limits the ability to capture more granular variations of IP in our sample. This approach was adopted post hoc to address smaller subgroup sample sizes and enable statistically meaningful analysis. While this allowed broader patterns to emerge, it may have oversimplified diversity within these categories and underrepresented the intersectional nature of identity and its influence on IP.

Our sample is not fully representative of all demographic groups in T&O, limiting the generalisability of this study. Nearly half of the sample (45%) was concentrated in specific regions, such as Yorkshire & Humber, the Northwest of England and the West Midlands. The response rate was 8.5%, which constitutes a key limitation and potential source of response bias. The sensitive nature of IP as a psychological construct may have contributed to this, as stigma around acknowledging self-doubt in high-achieving professionals may deter participation. Despite this limitation, our prevalence estimates are consistent with recent US-based studies in orthopaedic surgery, and the observed gender disparities align with international findings. This concordance suggests that, although the sample may be self-selecting, the patterns identified are likely to reflect broader experiences within the specialty. However, the limited sample size precluded the analysis of the association between gender and IP severity across different training levels; this remains an important area for further investigation.

Furthermore, although the impact assessment was designed based on the existing literature, this approach may introduce bias by predefining the domains rather than allowing participants to identify them independently. However, this framework was necessary to explore whether IP impacts similar domains in T&O and to highlight areas for future research.

### Scope for future research

Future research must also delve into intersectionality, examining how factors such as gender, ethnicity and other demographics shape IP experiences. Adopting more nuanced approaches to these variables could elucidate the role of systemic biases, stereotyping and exclusion tied to racialised identities and workplace dynamics. Given the gaps identified in our sample, a qualitative study focusing on female surgeons and extending to other underrepresented ethnic groups could provide richer insights into how IP is experienced across intersecting identities and career stages in T&O.

A significant knowledge gap also persists regarding IP’s influence on leadership and patient care. Investigating how IP affects decision-making, leadership confidence and team dynamics in high-stakes clinical settings is critical. Such studies could explore whether hesitation or self-doubt linked to IP impacts patient outcomes and team cohesion, emphasising the need for targeted interventions to address these challenges.

## Conclusion

This study provides an analysis of IP among T&O surgeons in the UK, highlighting its high prevalence and impact on leadership domains in the speciality. IP is particularly pronounced among female surgeons, trainees and those returning after career breaks, underscoring the systemic barriers that hinder the development of diverse and inclusive leadership within T&O surgery. The findings highlight that moderate to intense IP undermines confidence, decision-making and the pursuit of leadership roles. To improve diversity in leadership in T&O, IP must be addressed using a multifaceted approach, including individual support to reframe self-doubt, peer-to-peer mentorship to build confidence and institutional initiatives to address cultural and systemic factors that perpetuate IP.

## Data Availability

Data are available upon reasonable request.
