# Supplementary material for: Hidden barriers to leadership: a cross-sectional survey of prevalence and predictors of Imposter Phenomenon in Trauma and Orthopaedic surgery in the UK
Source: BMJ Open. 2025 Sep 11;15(9):e100557. doi: 10.1136/bmjopen-2025-100557 (PMC12519336; doi:10.1136/bmjopen-2025-100557)
Supplement: Supplementary file 1 [file bmjopen-2025-100557.reviewer_comments.pdf]

PEER REVIEW HISTORY

BMJ Open publishes all reviews undertaken for accepted manuscripts. Reviewers are asked to complete a checklist review form and are provided with free text boxes to elaborate on their assessment. These free text comments are reproduced below.

ARTICLE DETAILS

Title (Provisional)

Hidden Barriers to Leadership: A Cross-Sectional Survey of Prevalence and Predictors of Imposter Phenomenon in Trauma and Orthopaedic Surgery in the UK

Authors

Kamran Siddiqui, Zaha; Tomlinson, James; Scantlebury, Arabella; Jayasuriya, Raveen; Church, Helen; Grove, Amy

---

VERSION 1 - REVIEW

**Reviewer**	**1**
**Name**	**Weaver, Jessica**
**Affiliation**	**UC San Diego**
**Date**	**13-May-2025**
**COI**	**None**

Thank you for the opportunity to review this manuscript. The authors present a cross-sectional study describing rates and severity of imposter phenomenon among Trauma and Orthopedic surgeons across the UK. Perhaps most importantly they demonstrate that frequent or intense imposter syndrome symptoms are common across gender and ethnic divides. The methods and results are well-described and the discussion is well-written. I have a few minor comments:

1) Under the Methods Analysis section where the different ethnicity categories are described, there is an opening parenthesis missing after “people of color.” Also, were people not given the option to indicate ethnic origins from Central or South America, or were there just no respondents from this area?

2) In the background, the authors describe how most publications examining IP are from the US, as well as a few from other countries, but that this was the first study looking specifically in the UK. How do the results in the UK compare to those in other countries? If there are any differences, are there any cultural reasons or any unique aspects of medical training/practice in the UK that explain any differences?

3) The authors note that women have higher rates of imposter syndrome, and discuss that this may inhibit career advancement. If possible, it would be interesting to look at the rates/severity of imposter syndrome specifically in women at different levels of training to see if the severity of IP changes with career advancement.

**Reviewer**	**2**
**Name**	**Wadhwa, Harsh**
**Affiliation**	**Stanford University**
**Date**	**22-May-2025**
**COI**	**None**

Congratulations to the authors on a well-designed and conducted study.

A few comments and suggestions below:

Please outline all demographic information used as potential predictors of imposter phenomenon in the methods section.

While bias was addressed in the limitations, the overall small response rate (8.5%) must be highlighted as a key limitation to this study, as this may be a source of response bias.

It would be beneficial to compare results to your counterparts in the US, as a recent study has similarly approached this topic for US trainees: Wadhwa H, Khela HS, Khela MS, Van Rysselberghe N, Hunt AA, Lu L, Bishop J. Imposter Syndrome Among Orthopaedic Surgery Residents is Extremely Common and Disproportionately Affects Female Residents. JB JS Open Access. 2025 Apr 7;10(2):e24.00132. doi: 10.2106/JBJS.OA.24.00132.

**Reviewer**	**3**
**Name**	**Peng, Jianan**
**Affiliation**	**Acadia University, Math and Stats**
**Date**	**21-Jul-2025**
**COI**	**None**

Hidden Barriers to Leadership: A Cross-Sectional Survey of Prevalence and Predictors of Imposter Phenomenon in

Trauma and Orthopaedic Surgery in the UK

Manuscript bmjopen-2025-100557

The manuscript is well written. Just minor revision regarding statistical analysis. Please specify the procedure names in SPSS on page 8. Please give some specific examples how to use Mann-Whitney U test to evaluate CIPS scores by gender and Kruskal-Wallis test for CIPS scores by training grade. On page 12 a multiple linear regression was used but the manuscript does not indicate what predictors are used in the regression.

VERSION 1 - AUTHOR RESPONSE

Reviewer: 1<br>Dr. Jessica Weaver, UC San Diego	
Under the Methods Analysis section where the different ethnicity categories are described, there is an opening parenthesis missing after "people of colour." Also, were people not given the option to indicate ethnic origins from Central or South America, or were there just no respondents from this area?	Thank you for this observation. We have corrected the missing opening parenthesis in the Methods section for clarity. Regarding ethnic categorisation, participants were given an "Other" option to self-identify ethnic origins not listed, including those from Central or South America. However, no respondents from these backgrounds participated in the survey.
In the background, the authors describe how most publications examining IP are from the US, as well as a few from other countries, but that this was the first study looking specifically in the UK. How do the results in the UK compare to those in other countries? If there are any differences, are there any cultural reasons or any unique aspects of medical training/practice in the	Thank you for this helpful comment. We have now addressed this comparison in the opening of the Discussion section. with new evidence from a US-based study in Orthopaedics, which provides more relevant comparison than studies from other surgical specialties. Our analysis demonstrates remarkably similar prevalence rates when comparable severity thresholds are applied (UK: 63% vs US: 73% for significant/intense IP), suggesting consistent patterns across healthcare systems in the field.

UK that explain any differences?	
The authors note that women have higher rates of imposter syndrome and discuss that this may inhibit career advancement. If possible, it would be interesting to look at the rates/severity of imposter syndrome specifically in women at different levels of training to see if the severity of IP changes with career advancement.	We thank the reviewer for this insightful suggestion. Examining the rates and severity of IP among women at different training levels would indeed provide valuable insights into how IP experiences may evolve throughout career progression in T&O surgery. However, given the restrictions around our sample size, it was not possible to explore the association between gender and severity. We agree that this is an interesting observation and have now added it to our strengths and limitations section.<br><br>To draw conclusive findings about IP severity across training levels specifically among female surgeons, we would require adequate representation of women at each training grade. Given the well-documented underrepresentation of women in T&O surgery, particularly at senior levels, our current sample size may not provide sufficient power to detect meaningful differences between grades among female participants alone. We agree that this represents an important gap in the literature. A qualitative study exploring the role of IP in female surgeons and extending this to other underrepresented groups not reflected in our survey (e.g., individuals identifying as Central or South American), would be a valuable next step. Such work could provide richer insights into the intersection of IP, gender, ethnicity, and career progression in T&O. We have now noted this in the discussion as a potential direction for future research.
Reviewer: 2<br>Dr. Harsh Wadhwa, Stanford University	
Please outline all demographic information used as potential predictors of imposter phenomenon in the methods section.	The demographic information used as potential predictors of imposter phenomenon is already outlined in the Methods section under the subheading "Variables." Specifically, the text states: *"Predictors included demographic variables (e.g., gender, ethnicity, training grade, and time out of training)."*<br><br>To improve clarity, I have revised this statement to read: *"Predictors included demographic variables: gender, ethnicity, training grade, and time out of training."* This change explicitly lists the variables without parentheses or examples, making it clearer to the reader which demographic factors were considered.
While bias was addressed in the limitations, the overall small response rate (8.5%) must be	We thank the reviewer for this important observation. We acknowledge that our response rate of 8.5% is below the average response rate of 44.1% reported for physician surveys (Cho et al., 2013) - https://www.sciencedirect.com/science/article/pii/S245195882

highlighted as a key limitation to this study, as this may be a source of response bias.	2000409. However, we believe several factors provide context for interpreting these findings: the sensitive nature of Imposter Phenomenon may naturally result in lower response rates due to stigma around acknowledging self-doubt in high-achieving professionals. Importantly, despite the lower response rate, our findings demonstrate remarkable consistency with existing literature - our prevalence rates (63% for frequent/intense IP) closely align with the 73% prevalence reported in a recent US-based study of Orthopedic residents (Wadhwa et al., 2025), and our gender disparities mirror international findings. Additionally, our sample demonstrates geographical diversity across all UK regions and training levels, suggesting reasonable external validity.<br><br>We have strengthened the **limitations** section to explicitly address response bias concerns while providing context for the validity of our findings as above.
It would be beneficial to compare results to your counterparts in the US, as a recent study has similarly approached this topic for US trainees: Wadhwa H, Khela HS, Khela MS, Van Rysselberghe N, Hunt AA, Lu L, Bishop J. Imposter Syndrome Among Orthopaedic Surgery Residents is Extremely Common and Disproportionately Affects Female Residents. JB JS Open Access. 2025 Apr 7;10(2):e24.00132. doi: 10.2106/JBJS.OA.24.00132.	We thank the reviewer for highlighting this excellent study and congratulate the authors on their important contribution to understanding IP in orthopaedic surgery. We have referenced this study in our introduction, further incorporating detailed comparisons in the opening of our discussion section, and within the gender disparity subsection. This comparison has significantly strengthened our work by validating our findings through international replication (demonstrating remarkable alignment between UK (63%) and US findings (73%) for frequent - severe IP categories). Further highlighting the unique contributions of our study (career-wide analysis, time out of training as novel predictor) and demonstrating that IP in orthopaedic surgery may be driven by specialty-specific rather than system-specific factors.<br><br>**Please note that the inclusion of this study in our manuscript has resulted in updates to the in-text citations and reference list.**
Reviewer: 3<br>Dr. Jianan Peng, Acadia University	
The manuscript is well written. Just minor revision regarding statistical analysis. Please specify the	We are uncertain as to what the reviewer is asking for specifically, however we believe they would like to see the SPSS procedure used for analysis, which is copied below, it is not appropriate to include this in the manuscript.

procedure names in SPSS on page 8. Please give some specific examples how to use Mann-Whitney U test to evaluate CIPS scores by gender and Kruskal-Wallis test for CIPS scores by training grade.	In SPSS, the procedures used for non-parametric comparisons like Mann-Whitney U and Kruskal-Wallis are:<br>• **Mann-Whitney U Test**:<br>SPSS procedure: Analyze > Nonparametric Tests > Legacy Dialogs > 2 Independent Samples<br>• **Kruskal-Wallis Test**:<br>SPSS procedure: Analyze > Nonparametric Tests > Legacy Dialogs > K Independent Samples<br><br>**Mann-Whitney U Test for CIPS by Gender**<br>**Goal**: Compare **CIPS scores** between **male and female participants**.<br>**Steps in SPSS:**<br>1. Go to Analyze > Nonparametric Tests > Legacy Dialogs > 2 Independent Samples.<br>2. Move **CIPS score** to the **Test Variable List**.<br>3. Move **Gender** to the **Grouping Variable**.<br>4. Click **Define Groups** and enter group codes (e.g., 1 = Male, 2 = Female).<br>5. Make sure **Mann-Whitney U** is selected under **Test Type**.<br>6. Click **OK**.<br>**Hypothetical Example:**<br>• Variable: CIPS_Score<br>• Grouping variable: Gender<br>(coded as 1 = Male, 2 = Female)<br>**Interpretation**:<br>• If $p < 0.05$, there is a significant difference in CIPS scores between males and females.<br><br>**Kruskal-Wallis Test for CIPS by Training Grade**<br>**Goal**: Compare **CIPS scores** across multiple training grades (e.g., PGY1, PGY2, PGY3).<br>**Steps in SPSS:**<br>1. Go to Analyze > Nonparametric Tests > Legacy Dialogs > K Independent Samples.<br>2. Move **CIPS score** to the **Test Variable List**.<br>3. Move **Training Grade** to the **Grouping Variable**.<br>4. Click **Define Range** and enter minimum and maximum group codes (e.g., 1 to 3).<br>5. Ensure **Kruskal-Wallis H** is selected.<br>6. Click **OK**.<br>**Hypothetical Example:**<br>• Variable: CIPS_Score<br>• Grouping variable: Training_Grade<br>(coded as 1 = PGY1, 2 = PGY2, 3 = PGY3)<br>**Interpretation**:

	• If $p < 0.05$, at least one training grade group has a significantly different median CIPS score.
On page 12 a multiple linear regression was used but the manuscript does not indicate what predictors are used in the regression.	Thank you for your comment. The Results section on page 12 presents the predictors included in the multiple linear regression analysis, specifically consultant status, gender, time out of training, and ethnicity as predictors of CIPS scores. The results indicate that consultant status and male gender were significant negative predictors, time out of training had a significant positive effect, while ethnicity was not a significant predictor. No multicollinearity issues were detected.<br><br>To improve clarity, we have revised the Methods section to explicitly list these predictors on page 8 as follows:<br>*"A multiple linear regression analysis was conducted to assess potential predictors of IP severity. The predictors included demographic variables: consultant status, gender, ethnicity, and time out of training. Multicollinearity diagnostics were performed to ensure independence of predictor variables."*

VERSION 2 - REVIEW

**Reviewer**	**2**
**Name**	**Wadhwa, Harsh**
**Affiliation**	**Stanford University**
**Date**	**14-Aug-2025**
**COI**	

The authors have done a thorough job addressing the suggested revisions to the manuscript.

**Reviewer**	**3**
**Name**	**Peng, Jianan**
**Affiliation**	**Acadia University, Math and Stats**
**Date**	**16-Aug-2025**
**COI**	

Hidden Barriers to Leadership: A Cross-Sectional Survey of Prevalence and Predictors of Imposter Phenomenon in

Trauma and Orthopaedic Surgery in the UK

Manuscript bmjopen-2025-100557.R1

You do not need to copy SPSS procedure's menu. You can mention that Training_Grade is the group variable with 3 categories when you use Kruskal-Wallis Test for CIPS.
I am satisfied with the revision for page 12 regarding predictors.

VERSION 2 - AUTHOR RESPONSE

Reviewer: 3<br>Dr. Jianan Peng, Acadia University	
You do not need to copy SPSS procedure's menu. You can mention that Training_Grade is the group variable with 3 categories when you use Kruskal-Wallis Test for CIPS.	We thank the Dr Peng for this helpful clarification and guidance. We have revised the Analysis section to more clearly describe the statistical procedures and grouping variables.<br><br>Specifically, we now state that the Mann–Whitney U test was applied for two-group comparisons (gender: male/female; time out of training: yes/no), while the Kruskal–Wallis test was used for multi-group comparisons. For the latter, ethnicity was analysed as the grouping variable with three categories (POC, White, Other) and training grade as the grouping variable with two categories (consultants, non-consultants).<br><br>These revisions have been incorporated into the manuscript on pages 7–8. We trust this provides sufficient clarification of our analytical approach.
